# Nocturnal blood pressure dipping as a marker of endothelial function and subclinical atherosclerosis in pediatric-onset systemic lupus erythematosus

**DOI:** 10.1186/s13075-020-02224-w

**Published:** 2020-06-03

**Authors:** Joyce C. Chang, Rui Xiao, Kevin E. Meyers, Laura Mercer-Rosa, Shobha S. Natarajan, Pamela F. Weiss, Andrea M. Knight

**Affiliations:** 1 Division of Rheumatology, Children’s Hospital of Philadelphia, Philadelphia, PA 19104 USA; 2 Center for Pediatric Clinical Effectiveness, Children’s Hospital of Philadelphia Research Institute, Philadelphia, PA 19146 USA; 3 Department of Pediatrics, University of Pennsylvania Perelman School of Medicine, Philadelphia, PA 19104 USA; 4 Department of Biostatistics, Epidemiology and Informatics, University of Pennsylvania Perelman School of Medicine, Philadelphia, PA 19104 USA; 5 Division of Nephrology, Children’s Hospital of Philadelphia, Philadelphia, PA 19104 USA; 6 Division of Cardiology, Children’s Hospital of Philadelphia, Philadelphia, PA 19104 USA; 7 Center for Pharmacoepidemiology Research and Training, University of Pennsylvania, Philadelphia, PA 19146 USA; 8 Division of Rheumatology, Hospital for Sick Children, Toronto, ON Canada; 9 SickKids Research Institute, Hospital for Sick Children, Toronto, ON Canada

**Keywords:** Systemic lupus erythematosus, Pediatric systemic lupus erythematosus, Ambulatory blood pressure monitoring, Cardiovascular disease, Cardiovascular diagnostic techniques

## Abstract

**Background:**

Loss of the normal nocturnal decline in blood pressure (BP), known as non-dipping, is a potential measure of cardiovascular risk identified by ambulatory blood pressure monitoring (ABPM). We sought to determine whether non-dipping is a useful marker of abnormal vascular function and subclinical atherosclerosis in pediatric-onset systemic lupus erythematosus (pSLE).

**Methods:**

Twenty subjects 9–19 years of age with pSLE underwent ABPM, peripheral endothelial function testing, carotid-femoral pulse wave velocity/analysis for aortic stiffness, and carotid intima-media thickness. We assessed the prevalence of non-dipping and other ABPM abnormalities. Pearson or Spearman rank correlation tests were used to evaluate relationships between nocturnal BP dipping, BP load (% of abnormally elevated BPs over 24-h), and vascular outcome measures.

**Results:**

The majority (75%) of subjects had inactive disease, with mean disease duration of 3.2 years (± 2.1). The prevalence of non-dipping was 50%, which occurred even in the absence of nocturnal or daytime hypertension. Reduced diastolic BP dipping was associated with poorer endothelial function (*r* 0.5, *p* = 0.04). Intima-media thickness was significantly greater in subjects with non-dipping (mean standard deviation score of 3.0 vs 1.6, *p* = 0.02). In contrast, higher systolic and diastolic BP load were associated with increased aortic stiffness (*ρ* 0.6, *p* = 0.01 and *ρ* 0.7, *p* < 0.01, respectively), but not with endothelial function or intima-media thickness.

**Conclusion:**

In a pSLE cohort with low disease activity, isolated nocturnal BP non-dipping is prevalent and associated with endothelial dysfunction and atherosclerotic changes. In addition to hypertension assessment, ABPM has a promising role in risk stratification and understanding heterogeneous mechanisms of cardiovascular disease in pSLE.

## Background

Premature cardiovascular events remain the major cause of morbidity and mortality among patients with systemic lupus erythematosus (SLE) beyond the first several years of disease [1–3], and those with pediatric-onset SLE (pSLE) are at risk for cardiovascular events at even younger ages, between early to middle adulthood [4]. Existing non-invasive measures of cardiovascular risk in the pediatric population, such as carotid intima-media thickness (cIMT), have demonstrated accelerated progression of subclinical atherosclerosis in pSLE [5]. However, these methods are highly operator-dependent and primarily limited to research applications. As a result, there is a need for clinically feasible strategies to measure cardiovascular risk in pSLE at early stages when there is an opportunity to intervene.

**Fig. 1:**
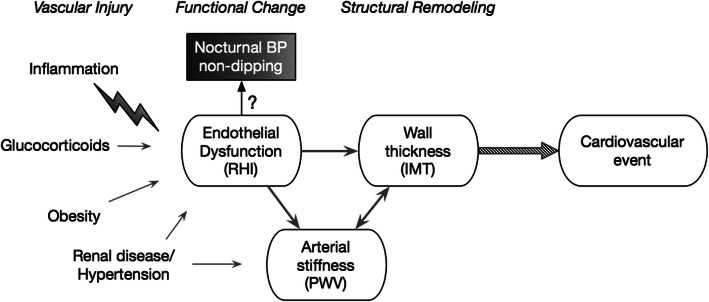
Conceptual model of the continuum of subclinical atherosclerosis and available measures of vascular health. Inflammatory states downregulate endothelium-dependent nitric oxide production and increase expression of adhesion molecules, leading to the inability to regulate vascular tone, cellular adhesion, and thrombosis that characterizes endothelial dysfunction. Subsequent maladaptive cellular changes result in increased arterial stiffness and thickening of the intimal-medial layers. RHI, reactive hyperemia index measures nitric oxide-dependent vasodilatory responses; PWV, pulse wave velocity and analysis approximate arterial stiffness; IMT, intima-media thickness by carotid ultrasound measures structural remodeling, which precedes plaque formation

Loss of nocturnal blood pressure (BP) dipping is a marker of increased cardiovascular risk that can be easily screened for using 24-h ambulatory blood pressure monitoring (ABPM). Normal blood pressure variability follows a diurnal pattern with a decline in BP at night. Loss of the physiologic nocturnal BP decline, also referred to as “non-dipping”, is an independent predictor of target organ damage and cardiovascular mortality in both hypertensive and normotensive adults [6–9]. This relationship is potentially mediated by endothelial dysfunction, one of the earliest stages of subclinical atherosclerosis (Fig. 1) [10]. Routine ABPM assessment of non-dipping is recommended in children with juvenile-onset diabetes [11], as it predicts progression to microalbuminuria [12]. To date, there are no specific guidelines on the use of ABPM in pSLE.

ABPM has several advantages over many research-based non-invasive measures of cardiovascular risk, because it is a clinically available tool with pediatric reference standards and does not suffer from operator-dependence. The magnitude of nocturnal BP dipping is normally distributed [13, 14], with a prevalence of non-dipping of 14% observed among non-obese children referred for ABPM for elevated casual BP [15]. Despite this, there is very limited data on the use of ABPM in pSLE. Campbell et al. first described ABPM abnormalities in a retrospective study of 10 children with pSLE and identified non-dipping in 90% [16]. However, these ABPM studies were clinically indicated and reflect the prevalence from targeted rather than routine screening. Furthermore, all subjects were taking glucocorticoids at the time of assessment. Canpolat et al. reported a prevalence of non-dipping of 67% in 24 pSLE subjects in Turkey, the majority of which were also still being treated with glucocorticoids and had active disease at the time of assessment [17]. The prevalence of non-dipping in an unselected US pSLE population and the contribution of glucocorticoid use have not been fully characterized. In addition, the clinical and physiologic relevance of non-dipping to early detection of cardiovascular disease in pSLE remains unknown.

Therefore, the objectives of this study were to (i) estimate the prevalence of nocturnal BP non-dipping in an unselected, racially diverse population of children and adolescents with pSLE; (ii) assess convergent validity between nocturnal BP dipping and other non-invasive measures of vascular health, including endothelial dysfunction, arterial stiffness and wall thickening (Fig. 1); and (iii) determine whether non-dipping status can be used to stratify early atherosclerotic risk.

## Methods

### Study design

This was a cross-sectional study of children and adolescents with pSLE who were prospectively recruited to undergo comprehensive non-invasive cardiovascular testing from October 2018 to June 2019.

### Study population

Subjects ages 9–21 years inclusive, meeting American College of Rheumatology or Systemic Lupus International Collaborating Clinics classification criteria for SLE by age 18, were recruited from the pediatric rheumatology clinic at a tertiary academic center. We excluded subjects with chronic kidney disease stage 3 or greater (estimated glomerular filtration rate (eGFR) < 60 mL/min/1.73 m^2^ for ≥ 3 months), on dialysis, after kidney transplantation, or with a history of obstructive sleep apnea, which are known to be independently associated with nocturnal hypertension. We also excluded subjects with known hypertension at enrollment, but patients with a history of resolved hypertension diagnosis were eligible for inclusion.

### Study measures

#### Blood pressure outcomes

##### Casual BP assessment

Trained research nurses measured casual BP using the auscultatory method at least twice in the right upper extremity while in the seated position with an aneroid sphygmomanometer (Mabis MedicKit 5; Mabis Healthcare, Waukegan, IL). If there was a discrepancy of more than 5 mmHg between two measurements in either systolic (SBP) or diastolic blood pressure (DBP), a third measurement was taken and the average of the three measurements was recorded.

##### Ambulatory BP assessment

Twenty-four-hour ABPM was performed using oscillometric Spacelab 90217 monitors (Spacelabs Medical, CA) according to American Heart Association guidelines for standard ambulatory assessment [18]. Systolic and diastolic BP were measured every 20 min while awake and every 30 min during sleep for 24 h. The primary outcome was the presence of non-dipping BP, defined as < 10% decrease from mean daytime to mean nighttime systolic or diastolic BP [19]. Secondary outcomes included masked hypertension (normal casual BP with abnormal mean 24-h BP or BP load), white coat hypertension (elevated casual BP with normal mean 24-h BP and BP load), nocturnal hypertension (> 25% of nighttime BP measurements above the 95th percentile for age, sex and height), and average 24-h systolic or diastolic BP load (total percentage of BP values above the 95th percentile) [13, 14, 19]. BP percentiles and ABPM hypertension category were classified based on the updated American Academy of Pediatrics clinical practice guidelines [11].

#### Measures of vascular function and stiffness

##### Peripheral endothelial function

Endothelial vasomotor function was assessed in the fasting state during morning hours in a temperature-controlled vascular lab using the EndoPAT device (Endo-PAT2000, Itamar-Medical, Caesarea, Israel), as previously described [20]. Digital pulse amplitude readings pre- and post-deflation were used to calculate the reactive hyperemia index (lnRHI), defined as the natural log transformation of the ratio of the post-deflation pulse amplitude over the baseline measurement in the hyperemic finger divided by the corresponding ratio in the control finger. LnRHI values ≤ 0.51 are considered abnormal [21].

##### Aortic stiffness

Carotid-femoral pulse wave velocity (PWV) was measured using the SphigmoCor Vx system (AtCor Medical Pty Ltd., Australia) [22]. PWV standard deviation scores (SDS) for age and sex were calculated using published reference values for mean transit velocity [23]. Pulse wave analysis (PWA) was also performed to calculate the aortic augmentation index, which approximates aortic stiffness.

#### Structural measure of subclinical atherosclerosis

##### Carotid intima-media thickness (IMT)

One experienced sonographer performed all high-resolution, real-time B-mode carotid ultrasound studies (ATL 3000). Serial images in longitudinal and transverse planes were obtained in the supine position at a 45° angle of insonation. Using edge-tracking software (EchoPAC PC, GE Medical Systems), an echocardiologist experienced in measuring carotid IMT (S.N.) performed three separate IMT measurements at the start of the R wave on the electrocardiograph (end-diastole) in the far wall of the right and left distal common carotid artery 10 mm proximal to the origin of the carotid bifurcation, the carotid bulb, and the internal carotid artery 10 mm distal to the bifurcation. The mean of the bilateral common carotid artery (CCA) measurements (CCA-IMT) and the mean of all 6 segments (mean cIMT) were used as the primary outcome measures of intima-media thickness [24, 25]. SDS by age and sex for CCA-IMT were calculated by the LMS method using published reference norms [26].

#### Covariates

##### Traditional cardiovascular risk factors

Subjects completed height and weight measurements for body mass index (BMI), a physical activity questionnaire [27], and a demographics survey (race, ethnicity, household income, parental education, family history of early cardiovascular disease). Each subject provided a fasting blood sample for measurement of lipids, high-sensitivity C-reactive protein by immunoassay (Roche Diagnostics c311), and lipoprotein A by enzyme-linked immunosorbent assay (Abcam 212165), which have previously been identified as risk factors for increased rates of atherosclerotic progression in SLE [5, 28]. Lipoprotein A values > 95th percentile for age and race/ethnicity (NHANES III) were considered abnormal [29].

##### Disease-related factors

Additional clinical data was abstracted from the electronic medical record, including disease duration, SLE manifestations, SLE Disease Activity Index (SLEDAI-2K) scores [30], glucocorticoid dose, immunosuppressive medication history, and the most recent serum creatinine and urine protein/creatinine levels. eGFR was calculated using the revised Schwartz equation for subjects ≤ 18 years of age and the Chronic Kidney Disease Epidemiology Collaboration equation for subjects > 18 years of age [31, 32]. Cumulative disease activity was calculated as a time-averaged mean [33] using historical abstractions of SLEDAI-2K scores from clinic visits. For subjects who never had hypocomplementemia or anti-dsDNA antibodies as a disease manifestation, the corresponding score component of zero was carried forward. We also retrospectively calculated the proportion of time in a lupus low disease activity state (LLDAS) to account for glucocorticoid dose [34].

### Statistical analysis

ABPM and other vascular outcome measures were summarized using standard descriptive statistics such as mean and standard deviation or median and interquartile range for continuous variables, and count and frequency for categorical variables. We used Shapiro Wilk’s test to assess for normality. A pre-specified significance level alpha of 0.10 (two-sided) was used for all analyses.

i) Prevalence of non-dipping and secondary ambulatory BP outcomes was calculated using the total number of adequate ABPM studies as the offset. To identify potential risk factors for non-dipping, differences in clinical characteristics by non-dipping status were assessed using Fisher’s exact, Student’s *t*, or Wilcoxon rank sum tests as appropriate.
ii) To assess convergent validity between the magnitude of nocturnal BP dipping and other measures of vascular health, we used Pearson correlation (*r*) coefficients. Since it is not known whether systolic or diastolic BP is more clinically relevant in SLE, systolic and diastolic BP dips were considered separately. We identified outliers using leverage plots and Cook’s distance and performed sensitivity analyses with and without influential outliers with a Cook’s distance > 0.22. In a secondary analysis, we used Spearman rank correlation (*ρ*) coefficients to evaluate the relationship between 24-h BP load and other vascular measures.
iii) To determine whether non-dipping is associated with atherosclerotic risk, we used two-sample *t* tests to test differences in CCA-IMT and mean cIMT between subjects with normal nocturnal dipping and those with non-dipping. To explore the discriminative ability of non-dipping with respect to high-risk CCA-IMT (SDS > 2.0), we assessed concordance using the C-statistic.

## Results

The mean age was 16.5 years (range 9–19) and the average disease duration was 3.2 years (± 2.1) (Table 1). Forty percent of subjects were African American. Seventy-five percent of subjects had inactive disease (SLEDAI-2K score < 4) at the time of enrollment. Twenty-five percent of subjects had a history of nephritis, of which only one had ongoing proteinuria and all had normal renal function (eGFR > 90 mL/min/1.73 m^2^).

**Table 1:** Clinical characteristics of pSLE subjects by nocturnal BP dipping status

	All subjects, *N* = 20	Normal, *N* = 9*	Non-dipping, *N* = 9	*p* value
Demographic characteristics				
Age in years, mean (± SD)	16.5 (± 2.7)	17.1 (± 2.2)	15.6 (± 3 .2)	0.24
Female sex, *n* (%)	17 (85)	7 (78)	8 (89)	1.00
Race				
White/Caucasian	7 (35)	4 (44)	2 (22)	0.61
Black/African American	8 (40)	2 (22)	5 (56)	
Asian	3 (15)	2 (22)	1 (11)	
Other race	2 (10)	1 (11)	1 (11)	
Hispanic ethnicity	3 (15)	1 (11)	2 (22)	1.00
Highest household education				
Did not complete high school	2 (10)	2 (22)	0 (0)	0.64
High school/general education	10 (53)	4 (44)	5 (56)	
Bachelor’s degree or more	7 (37)	3 (33)	3 (33)	
Low income household (< $25 k/year)	6 (30)	1 (12.5)	4 (44)	0.29
Traditional cardiovascular risk factors				
Physical activity score, mean (± SD)	1.9 (± 0.7)	1.8 (± 0.6)	1.9 (± 0.9)	0.83
BMI percentile for age-sex	68.2 (28.6)	56 (± 30.4)	81 (± 19.8)	0.05
Low-density lipoprotein (mg/dL)	90 (± 22)	90 (± 22)	88 (± 26)	0.91
High-density lipoprotein (mg/dL)	44 (± 5)	44 (± 5)	59 (± 8)	< 0.01
Triglycerides (mg/dL)	86 (± 43)	86 (± 43)	81 (± 30)	0.74
Elevated hsCRP, *n* (%)^§^	2 (10)	0 (0)	2 (22)	0.47
Elevated Lipoprotein A	2 (10)	0 (0)	1 (11)	1.00
Prior history of hypertension^	3 (16)	2 (22)	1 (11)	0.50
Family history early CVD	5 (25)	2 (22)	2 (22)	1.00
Disease characteristics				
Disease duration, years (± SD)	3.2 (± 2.1)	3.6 (± 2.3)	2.3 (± 1.8)	0.21
Most recent SLEDAI-2K, mean (± SD)^†^	2.9 (± 4.4)	2.6 (± 3.4)	2.9 (± 5.8)	0.88
Time-averaged SLEDAI	12.4 (± 7.3)	3.9 (± 2.1)	6.3 (± 4.6)	0.14
Proportion of time in LLDAS^‡^	0.57 (± 0.32)	0.67 (± 0.22)	0.45 (± 0.40)	0.16
Antiphospholipid antibodies, *n* (%)	6 (30)	4 (44)	2 (22)	0.62
Nephritis, *n* (%)	5 (25)	1 (11)	3 (33)	0.58
Urine protein to creatinine, median [IQR]^#^	0.1 [0.0–0.1]	0.1 [0.0–0.1]	0.1 [0.0–0.4]	0.56
Neuropsychiatric manifestation, *n* (%)	1 (5)	0 (0)	1 (11)	1.00
Current medication use				
Glucocorticoids, *n* (%)	5 (25)	1 (11)	4 (44)	0.29
Months of glucocorticoid use, median [IQR]	13 [3–20]	8 [2–30]	11 [4–19]	0.91
Hydroxychloroquine, *n* (%)	20 (100)	9 (100)	9 (100)	1.00
Mycophenolate	11 (55)	2 (22)	7 (78)	0.06
Methotrexate	5 (25)	3 (33)	2 (22)	1.00
Azathioprine	2 (10)	1 (11)	1 (11)	1.00
Rituximab (within last 12 months)	4 (20)	0 (0)	3 (33)	0.21
Renin-angiotensin system blocker	4 (20)	1 (11)	2 (22)	1.00
Other antihypertensive	2 (10)	1 (11)	0 (0)	1.00

Comparison of baseline clinical characteristics by non-dipping status using Fisher’s exact, Student’s *t* test or Wilcoxon rank sum test as appropriate

*Only 18/20 subjects completed ABPM wear to determine normal dipping vs non-dipping

^Previous hypertension diagnosis resolved by physician prior to enrollment

^§^High-sensitivity C-reactive protein > 3.0 mg/L

^†^SLEDAI < 5, low disease activity; 6–10, moderate; 11–19, high; maximum, 105

^‡^Lupus low disease activity state

^#^Random (spot) urine protein to creatinine ratio

ABPM was evaluable and well-tolerated in 18/20 subjects. Of the two subjects with insufficient ABPM data, one did not wish to be seen wearing the monitor at school, while the other was intolerant of cuff inflations. EndoPAT interpretation was precluded in two subjects, one due to vasculitic finger lesions resulting in poor waveforms, and another due to pre-pubertal age with small finger size, resulting in falsely low amplitudes. No subjects had active Raynaud’s at the time of assessment.

### Prevalence of non-dipping and other ambulatory BP abnormalities

The prevalence of non-dipping was 50%, which commonly occurred in the setting of otherwise normal ambulatory BP (Table 2). Two subjects (11%) had nocturnal hypertension, one of which also had non-dipping. All subjects who met criteria for non-dipping had reduced SBP dip, one of which also had reduced DBP dip. On average, the magnitude of nocturnal DBP dipping was greater (mean 17.6%, SD 5.7) compared to SBP dipping (mean 10.0%, SD 3.7). Agreement between hypertension class by casual BP measurement and ambulatory BP was poor (observed agreement 56%), with 6 cases of white coat hypertension and one case of masked hypertension (Table 2). There were no significant differences in 24-h BP load among non-dippers compared to those with normal BP dipping (mean SBP load 4% vs. 12%, *p* = 0.18; mean DBP load 8% vs. 11%, *p* = 0.96). There was also no association between the magnitude of SBP dipping and nocturnal SBP load (*ρ* = 0.04, *p* = 0.88) or DBP dipping and nocturnal DBP load (*ρ* = − 0.29, *p* = 0.24).

**Table 2:** Presence of non-dipping BP in pSLE despite otherwise normal ABPM

	Adequate ABPM studies, *N* = 18	Non-dipping BP, *N* = 9
Normal, *n* (%)	10 (55%)	5 (56%)
White coat hypertension	6 (33%)	3 (33%)
Masked hypertension	1 (6%)	0 (0%)
Unclassified*	1 (6%)	1 (11%)

Blood pressure categories based on updated definitions from the 2017 AAP guidelines

Nocturnal hypertension: SBP/DBP load ≥ 50% by ABPM

White coat hypertension: Casual BP ≥ 95th percentile but normal ABPM (mean 24-h SBP/DBP < 95th percentile and SBP/DBP load < 25%)

Masked hypertension: normal clinic BP but mean 24-h SBP/DBP > 95th percentile and SBP/DBP load ≥ 25% by ABPM

*Unclassified: elevated clinic BP (> 90th but < 95th percentile) with normal ABPM

### Patient characteristics associated with non-dipping BP pattern

Subjects with non-dipping BP had higher mean BMI (mean 81st percentile vs. 56th percentile, *p* = 0.05) and greater high-density lipoprotein levels (mean 59 (± 8) vs. 44 (± 5) mg/dL, *p* < 0.01) compared to subjects with normal BP dipping (Table 1). Forty-four percent of non-dippers were still using glucocorticoids compared to 11% of subjects with normal dipping (*p* = 0.29). The remaining 56% of non-dippers had discontinued all glucocorticoids a median of 367 days (range 308–1367) before the study visit. The mean time-averaged cumulative disease activity was 6.3 (± 4.6) among non-dippers compared to 3.9 (± 2.1) among subjects with normal dipping, although the difference was not statistically significant (*p* = 0.14).

### Association between nocturnal BP dipping and vascular function and stiffness

#### Peripheral endothelial function

The prevalence of endothelial dysfunction was 22% (Table 3). There was a significant association between greater magnitudes of DBP dipping and better endothelial function (*r* 0.5, *p* value 0.04, *n* = 18). In contrast, BP load did not correlate with endothelial function (Table 4).

**Table 3:** Summary of vascular outcome measures in pSLE subjects

	*n*	Mean	SD	Abnormal, *n* (%)
lnRHI*	18	0.74	0.24	4 (22%)
PWV, m/s	20	4.81	0.93	
PWV SDS for age/sex^‡^	20	− 0.75	1.29	1 (5%)
Augmentation index, %	20	0.9	13.7	
CCA-IMT, mm	20	0.500	0.056	
CCA-IMT SDS for age/sex^†^	20	2.36	1.26	12 (60%)

*Reactive hyperemia index; lnRHI ≤ 0.51 was considered abnormal; waveforms were uninterpretable in 2 subjects

^‡^Pulse wave velocity standard deviation scores for age and sex derived from published reference norms; SDS > 2.0 was considered abnormal

^†^Common carotid intima-media thickness standard deviation scores derived from published references norms; SDS > 2.0 was considered abnormal

**Table 4:** Magnitude of nocturnal BP dipping correlates with vascular function and subclinical atherosclerosis

Measure	*N*	% SBP Dip		% DBP Dip		SBP load		DBP load	
		*r*	*p* value	*r*	*p* value	*ρ*	*p* value	*ρ*	*p* value
Aortic stiffness									
PWV SDS	18	0.3	0.29	− 0.1	0.76	0.3	0.28	0.0	0.87
Augmentation index	18	0.3	0.30	0.3	0.31	0.6	0.01	0.7	< 0.01
Endothelial function									
lnRHI	16	0.2	0.49	0.5	0.04	− 0.2	0.50	− 0.2	0.44
Wall thickening									
CCA-IMT	18	− 0.5	0.06	− 0.2	0.49	− 0.1	0.62	− 0.3	0.27
Mean cIMT	14	− 0.6	0.03	− 0.1	0.72	− 0.2	0.51	− 0.1	0.79

Pearson’s correlation coefficients (*r*) estimating the relationship between the percent dip in nocturnal BP and measures of vascular function and subclinical atherosclerosis; Spearman rank correlations (*ρ*) were used to assess associations with 24-h BP load

*SBP* systolic blood pressure, *DBP* diastolic blood pressure, *PWV SDS* pulse wave velocity standard deviation score for age and sex, *lnRHI* reactive hyperemia index, *CCA-IMT* mean common carotid intima-media thickness, *Mean cIMT* mean of IMT measurements over 6 carotid sites

#### Aortic stiffness

Abnormal PWV (SDS > 2) was observed in only one subject with an inadequate ABPM study. Mean augmentation index was 0.9 (± 13.7). Higher estimates of aortic stiffness by augmentation index, but not PWV, were strongly correlated with both 24-h SBP and DBP load (*ρ* 0.6 and 0.7, respectively, *p* values ≤ 0.01). In contrast to endothelial function, there was no significant correlation between SBP/DBP dipping and measures of aortic stiffness (Table 4).

### Non-dipping as a marker of subclinical atherosclerosis

#### Carotid intima-media thickness

The average CCA-IMT SDS was 2.36 standard deviations above the norm for age and sex (Table 3). Non-dipping BP pattern was associated with statistically significant increases in IMT across all carotid segments evaluated (Table 5). This difference was primarily driven by changes in systolic dip. A lower magnitude of SBP dipping correlated with significantly greater CCA-IMT (*r* − 0.47, *p* value 0.06) and ICA-IMT (*r* − 0.58, *p* value 0.03) (Table 4). In contrast, there was no significant correlation between the magnitude of DBP dipping and CCA-IMT (*r* − 0.17, *p* value 0.49). There were also no significant associations between SBP or DBP load and IMT outcome measures (Supplemental Table).

**Table 5:** Intima-media thickness is greater in pSLE subjects with non-dipping BP

	Normal, *N* = 9	Non-dipping, *N* = 9	*p* value*
Common carotid (CCA-IMT)	0.466 (0.048)	0.525 (0.049)	0.020
SDS for age/sex^†^	1.6 (1.1)	3.0 (1.2)	0.015
Carotid bulb-IMT	0.438 (0.061)	0.511 (0.080)	0.047
Internal carotid (ICA-IMT)^	0.387 (0.039)	0.472 (0.081)	0.023
Mean cIMT (6 sites)^	0.423 (0.038)	0.488 (0.069)	0.042

*Comparison of absolute cIMT (mm) and standard deviation scores (SDS) by non-dipping status using Student’s *t* test

^†^Standard deviation scores for age and sex derived from published references norms (Doyon et al.)

^Only *n* = 14 evaluable studies

CCA-IMT was abnormally increased (SDS > 2.0) in 60% of the total cohort and 56% of those with adequate ABPM studies. Non-dipping BP was observed in 7/10 subjects with increased CCA-IMT SDS compared to 2/8 subjects with normal CCA-IMT SDS, corresponding to a C-statistic of 72% of subjects correctly classified.

## Discussion

In this study of comprehensive pSLE cardiovascular profiles, there are several important findings that inform the utility and interpretation of ABPM in pSLE. First, systolic non-dipping was significantly associated with increased carotid IMT, which supports the potential role for ABPM in assessment of atherosclerotic risk in this population. Second, decreased diastolic BP dipping was associated with poorer endothelial function, suggesting that ABPM may reveal information about heterogeneous mechanisms of cardiovascular risk in pSLE. Lastly, non-dipping BP patterns were common in this cohort of pSLE subjects despite low disease activity, and these findings were independent of hypertension and glucocorticoid use. This study supports the potential value of utilizing ABPM to assess cardiovascular health in this high-risk population.

In support of the relevance of isolated non-dipping to cardiovascular risk in pSLE, we observed that loss of systolic BP dipping was the single ABPM measurement that was most strongly associated with structural evidence of subclinical atherosclerosis by cIMT. This suggests that there may be important clinical implications of non-dipping even in normotensive children and adolescents with pSLE. In addition, non-dipping may be particularly useful as a screening tool in pSLE because it is clinically feasible and less operator-dependent than cIMT. Higher frequencies of non-dipping (31–40%) have also been observed in pediatric obesity [15, 35]. Although obesity may contribute to the relationship between non-dipping and increased cIMT in our study [36, 37], a similar prevalence of non-dipping and association with cIMT was reported in a Turkish cohort of pSLE patients who had normal BMI [17], and therefore obesity is unlikely the only explanation for our findings. Larger ABPM studies will be needed to distinguish direct and indirect effects of disease-specific factors and potential confounders such as obesity. In addition, in this primarily normotensive cohort of pSLE subjects, non-dipping was a better predictor of increased cIMT than 24-h blood pressure or BP load. Further investigation is warranted to determine whether non-dipping in pSLE patients is independently prognostic of worse cardiovascular outcomes.

In addition to structural changes of subclinical atherosclerosis, nocturnal BP dipping patterns may also provide information about endothelial function. In our study, lower magnitudes of diastolic BP dipping correlated with worse peripheral endothelial function by digital reactive hyperemia. Associations between non-dipping and endothelial function have also been observed in pediatric hematopoietic cell transplant patients [38] and adults with resistant hypertension [39]. Furthermore, abnormal digital reactive hyperemia by EndoPAT has been shown to be predictive of cardiovascular events in adults [40], especially non-obstructive coronary events in women mediated by coronary microvascular dysfunction [41]. Therefore, the lack of concurrent association between diastolic BP dipping and cIMT (as initially hypothesized in Fig. 1) could potentially be explained by a more direct pathway from endothelial dysfunction to non-obstructive coronary disease, independent of atherosclerotic burden. Endothelial dysfunction has previously been proposed as a pathophysiologic mechanism of the association between non-dipping and cardiovascular disease [42–44]. Studies in adults with SLE suggest that high IFNα expression impairs nitric oxide release, which promotes endothelial dysfunction and supports the biologic plausibility of this mechanism of cardiovascular risk in SLE [45, 46]. More importantly, both endothelial function and the cardiovascular risk associated with non-dipping have been shown to be modifiable in other conditions, including rheumatoid arthritis and hypertension, respectively [47, 48], demonstrating reversibility of these measures and potential opportunities for intervention in SLE. This highlights the need for further studies of the longitudinal relationships between non-dipping and endothelial function in pSLE with cardiovascular outcomes such as non-obstructive coronary artery disease.

Our study also provides evidence that secondary risk factors such as hypertension, renal disease and glucocorticoid use are not sufficient to explain the high prevalence of non-dipping in pSLE. Non-dipping was frequently an isolated finding and independent of both systolic and diastolic BP load. This suggests that in chronic inflammatory states such as pSLE, non-dipping is less likely to be on a simple continuum with nocturnal or daytime hypertension. Chronic kidney disease is also a known independent risk factor for non-dipping [49]; however, the majority of non-dippers in our study either never had renal involvement or had inactive nephritis with normal renal function. Therefore, ABPM may have an important role in pSLE even in the absence of known renal involvement. Disruptions in diurnal BP patterns are hypothesized to result from dysregulation of neurohumoral regulatory pathways that follow circadian rhythms, providing qualitatively different information than overall elevations in BP [50]. Glucocorticoids may blunt diurnal variation by disrupting the hypothalamic-pituitary adrenal (HPA) axis. However, the majority of pSLE subjects with non-dipping in our study had discontinued glucocorticoids a year or more prior, and therefore disruption of the HPA axis by glucocorticoids is also insufficient to explain the prevalence of non-dipping in pSLE.

Lastly, while further investigation is required to determine the role of nocturnal BP dipping for cardiovascular health assessment in pSLE, ABPM has clear utility for hypertension assessment. The agreement between ambulatory and casual BP measurements in this study was extremely poor, with one case of masked hypertension resulting in initiation of antihypertensive treatment, as well as 6 cases of white coat hypertension. Of note, all of the study visits started in the early morning, so the discrepancy between ambulatory and casual BP could have been inflated by morning blood pressure surge [51], which has yet to be studied in children. Measurement of ambulatory blood pressure in pSLE provides a more accurate assessment of cardiovascular risk related to hypertension and should be considered for routine screening in addition to office-based casual BP measurement.

Strengths of this study include the use of multimodality vascular assessments protocolized for research purposes, which contributes to our understanding of the physiologic relevance of non-dipping. This is the first study to assess peripheral endothelial function by digital reactive hyperemia in pSLE, which is well-tolerated and more easily reproducible than operator-dependent methods such as brachial artery reactivity by flow-mediated dilation [52]. In addition, the racial, ethnic, and socioeconomic diversity of this study cohort contribute to the generalizability of our findings. There are also several limitations. Due to the small sample size and cross-sectional design, we were unable to perform multivariable adjustments or assess for effect modification. In addition, although 24-h ABPM was well-tolerated in most cases, we did not directly assess sleep quality, and therefore the impact of sleep on ABPM measurements will need to be accounted for in future studies. With respect to EndoPAT technology, readings are not interpretable in pSLE patients with active Raynaud’s or digital vasculitis. Falsely low readings have also been reported in young children due to the one size fit all probe [52], and therefore separate validation studies would need to be conducted before application of this method in rheumatic conditions that affect pre-pubertal children.

## Conclusions

In summary, loss of nocturnal BP dipping is common even in pSLE patients without clinically suspected hypertension and may serve as a useful surrogate marker of early vascular dysfunction and subclinical atherosclerosis. Our findings suggest that attenuation of either systolic or diastolic BP dipping may be indicative of different pathologic cardiovascular states in pSLE. With the added utility for hypertension assessment, ABPM could potentially be used to screen for multiple mechanisms of cardiovascular risk. Larger longitudinal studies are warranted to determine whether non-dipping is predictive of vascular disease progression, and whether it may serve as a potential therapeutic target in pSLE. In addition, further investigation of the pathways through which pSLE and its comorbidities influence ABPM measures is needed to inform the development of guidelines for ABPM use and interpretation in pSLE.

## Supplementary information

**Additional file 1: Supplemental Table.** Correlation between daytime or nighttime BP load and vascular measures.

## Data Availability

The data that support the findings of this study are available on request from the corresponding author (J.C.). The data are not publicly available due to information that could compromise research participant privacy.

## Abbreviations

BP: Blood pressure
pSLE: Pediatric-onset systemic lupus erythematosus
ABPM: Ambulatory blood pressure monitoring
cIMT: Carotid intima-media thickness
SBP: Systolic blood pressure
DBP: Diastolic blood pressure
lnRHI: Natural log of the reactive hyperemia index
PWV: Pulse wave velocity
SDS: Standard deviation score
PWA: Pulse wave analysis
CCA: Common carotid artery
ICA: Internal carotid artery
BMI: Body mass index
SLEDAI-2K: Systemic lupus erythematosus disease activity index
LLDAS: Lupus low disease activity state
