# Supplementary material for: Nocturnal blood pressure dipping as a marker of endothelial function and subclinical atherosclerosis in pediatric-onset systemic lupus erythematosus
Source: Arthritis Res Ther. 2020 Jun 3;22:129. doi: 10.1186/s13075-020-02224-w (PMC7268394; doi:10.1186/s13075-020-02224-w)
Supplement: Supplementary file 1 — Additional file 1: Supplemental Table. Correlation between daytime or nighttime BP load and vascular measures. [file 13075_2020_2224_MOESM1_ESM.docx]

| Supplemental Table. Correlation between daytime or nighttime BP load and vascular measures | | | | | | | | | |
| --- | --- | --- | --- | --- | --- | --- | --- | --- | --- |
|  |  | Systolic BP Load | | | | Diastolic BP Load | | | |
|  |  | Day | | Night | | Day | | Night | |
|  | N | ρ |  | ρ |  | ρ |  | ρ |  |
| PWV SDS for age | 18 | 0.29 |  | 0.18 |  | 0.02 |  | 0.06 |  |
| Augmentation index | 18 | 0.58 | ** | 0.41 | * | 0.73 | *** | 0.51 | ** |
| lnRHI | 18 | -0.26 |  | -0.37 |  | -0.10 |  | -0.15 |  |
| CCA-IMT | 18 | -0.13 |  | -0.05 |  | -0.40 |  | -0.04 |  |
| Carotid bulb-IMT | 18 | -0.06 |  | -0.11 |  | -0.42 |  | -0.19 |  |
| ICA-IMT | 14 | -0.28 |  | -0.21 |  | -0.10 |  | -0.02 |  |
| Mean cIMT (6 site) | 14 | -0.25 |  | -0.21 |  | -0.17 |  | -0.11 |  |
| Spearman rank correlations between BP load and measures of vascular function and structure | | | | | | | | | |
| * p-value < 0.10; ** p-value < 0.05, *** p-value < 0.01 | | | | | | | | | |
| PWV = pulse wave velocity; SDS = standard deviation score; lnRHI = natural log-transformation of reactive hyperemia index; CCA-IMT = common carotid artery intima media thickness; ICA = internal carotid artery | | | | | | | | | |
